# Adhesive Bonding of Copper Prepared by Laser-Interference near the Interference Structuring Limits

**DOI:** 10.3390/ma14133485

**Published:** 2021-06-23

**Authors:** Haotian Liu, Justin A. Weibel, Adrian S. Sabau, Patrick Geoghegan, Jian Chen, Eckhard A. Groll

**Affiliations:** 1 School of Mechanical Engineering, Purdue University, West Lafayette, IN 47906, USA; jaweibel@purdue.edu (J.A.W.); groll@purdue.edu (E.A.G.); 2 Computational Sciences & Engineering Division, Oak Ridge National Laboratory, Oak Ridge, TN 37831, USA; sabaua@ornl.gov; 3 Chemical, Biological & Environmental Engineering, Oregon State University, Corvallis, OR 97331, USA; patrick.geoghegan@oregonstate.edu; 4 Materials Sciences & Technology Division, Oak Ridge National Laboratory, Oak Ridge, TN 37831, USA; chenj2@ornl.gov

**Keywords:** laser, interference, adhesive, joining, copper

## Abstract

Adhesive bonding requires adequate surface preparation for ensuring an appropriate joint quality. The interest in adhesive joining has recently expanded to thermal systems having a large number of joints employed for manufacturing and assembly. This study presents surface topology of copper 110 produced by a laser-interference setup that would theoretically yield a periodicity of 1.7 μm, which is near the 1.6–2 μm structuring limit that was estimated based on thermal diffusion length scale for an 8 ns laser pulse. The results show that although the expected periodic interference structuring was not attained, the melt-induced texturing was affected by the laser-interference profile. Single-lap shear tests were performed with specimen surfaces prepared by traditional abrasion and laser-interference structuring methods. Several laser processing parameters, such as the laser spot size, density, number of pulses, and raster speed, were studied. Scanning electron microscope and profilometry measurements were used to characterize the processed surface microstructures. Web-like structures, which indicate widespread melting, were shown to be formed at different processing conditions. Based on the surface topologies investigated, two laser raster speeds were selected to make single-lap-joint specimens. Baseline joints were prepared by abrading joining specimens. The shear-lap strength and displacement at maximum load were shown to be higher by 16.8% and 43.8% for the laser-structured specimens than those of the baseline specimens, respectively. Moreover, the load-displacement curves indicated that the laser-structured joints were more ductile than those without laser-structuring. The increased ductility for the laser-structured joints was found to yield an increase in the energy absorbed during shear-lap testing of approximately of 80–90% over those measured for baseline joints. It is another indicator that laser-interference structuring enhanced the bonding performance of single-lap shear joints.

## 1. Introduction

Adhesive joining is an attractive technology that has been widely adopted in automotive and aerospace industries. Recently, there is an increasing need for alternative joining technology for component and system manufacturing in the HVAC&R (heating, ventilation, air conditioning, and refrigeration) industry. For the HVAC&R industry, adhesive joining has potential energy and cost savings benefits, especially if the mechanical properties of the adhesive joints would be similar or better than those of traditional brazed joints [1]. For the components and tubes in HVAC&R systems, copper and its alloys are the most common materials. However, Cu reacts with the atmospheric oxygen and forms an oxide surface layer. The oxide layer, together with oils and other contaminants on the surface, reduces the surface energy and bonding strength [1,2,3]. Thus, especially for Cu, surface preparation for joining is necessary and critical to ensure the bond quality and performance. The most common surface preparation techniques employed in industry include chemical and mechanical methods. Chemical methods [1,4,5] usually involve chemical etching or cleaning with solvents, which are toxic or flammable, such as strong acids, acetone, or isopropyl alcohol (IPA). Mechanical methods [4,5] usually involve sanding, blasting, or brushing. These traditional methods are labor intensive and costly to ensure quality control, environmental protection, and management of the hazardous materials.

In recent decades, with the development of laser technologies, new surface preparation techniques using high-energy lasers have attracted research interest. Compared with traditional methods, laser processing is a single-step and non-contact method. Laser processing has been shown to achieve surface cleaning and structuring. Previous research has studied the laser surface preparation of various materials with different laser types, energy levels, and optical setups. Neddersen et al. [6] showed that for the activity of surface-enhanced Raman scattering colloids, laser ablation was comparable or superior to that of chemical preparation. The surface morphologies produced by laser processing have been studied for different materials, such as Cu alloys, Al alloys, Ti alloys, or carbon fiber polymer composites (CFPC), using different laser processing methods and parameters [7,8,9,10,11].

The bonding performance enhancement by laser surface preparation has also been studied for Al alloys, Ti alloys, and polymers, and it was shown that laser structuring could increase the bonding performance [2,10,11]. Baburaj et al. [7] studied a laser ablation process that generates micro-columnar arrays and increases the adhesive bonding strength between plates by several mechanisms. Three reasons were found for bond strength improvement: increase in bonding surface area, mechanical locking of adhesive between micro-columns, and modifications in surface chemistry of the adherent that improved the surface wettability. Romoli et al. [12] studied the influence of surface laser texturing on adhesive strength using cylindrical specimens of aluminum alloys and showed an improvement of nearly 30% with respect to those of non-treated specimens. Hernandez et al. [13] analyzed the effect of pulsed laser ablation on copper substrates for adhesive bonding by using scanning electron microscopy (SEM), X-ray photoelectron spectroscopy (XPS), and finite element simulations. Results indicated that the laser ablation modifies surface morphology and chemistry, enabling enhanced mechanical interlocking.

This paper explores use of the laser-interference structuring technique [14,15,16] of copper for joining of HVAC components. Sabau et al. [17,18] characterized the surface morphology and structures produced by different laser fluence using Al alloy, CFPC, and conducted single-lap shear testing [19]. It was concluded that the laser-interference technique yielded periodic structuring on the surfaces with a significant increase in the bonding strength. A similar bonding enhancement effect was also noted for laser-interference structuring of AlMg3 and Ti6Al4V with woven hybrid yarn composites of glass fiber/polypropylene [20]. Compared to simple one-single beam laser ablation, the laser-interference technique creates periodic arrays on metallic surfaces in a size range from sub-micrometer to micrometers [14,15,16]. Laser-interference techniques split the main laser beam into two (or more) beams and then guide the separate laser beams back to the targeted area using optical components. When the laser beam hits the metal surface, the dominating free-electron cloud on the surface will quickly transfer the electromagnetic wave into phonons. The subsequent photothermal effect creates a surface heat treatment with a designed spatial power distribution, with hot and cold spots along the surface according to the wave interference. Based on the designed interference, different patterns can be achieved, such as dot-, line- and ring-shaped microstructures. The first laser-interference structures were reported in 1965 by Birnbaum [21], who used ruby lasers to produce regular patterns on semi-conductors. While initial studies were focused on the proof-of-principle of laser-interference structuring employing a spot-by-spot technique, i.e., firing several laser pulses while the laser beams were held focused on the same spot [15], more recent studies employed a raster technique [22], in which the laser spot moves at a constant speed while the laser is on.

After the description of the experimental setup and procedures for laser-interference surface preparation of copper, the surface profile using SEM and profilometry is presented. Adhesive joining is then presented. Single-lap shear tests were performed. The bonding performance of laser-structured surfaces was tested, analyzed, and compared to an abrasion-based surface preparation method, which is considered as a baseline for the current state-of-the-art.

## 2. Materials and Methods: Surface Preparation, Characterization, Joining, and Mechanical Testing Procedures

Copper 110 was selected, as it is commonly used in the HVAC industry. All of the specimens were cut from the same batch of material without any additional surface preparation or cleaning prior to the laser processing step. The specimens were cut in equal sizes of 101.6 mm (4 in.) by 25.4 mm (1 in.), as required for the single-lap shear testing.

### 2.1. Laser-Interference Technique and Target Surface Topology

**Figure 1:**
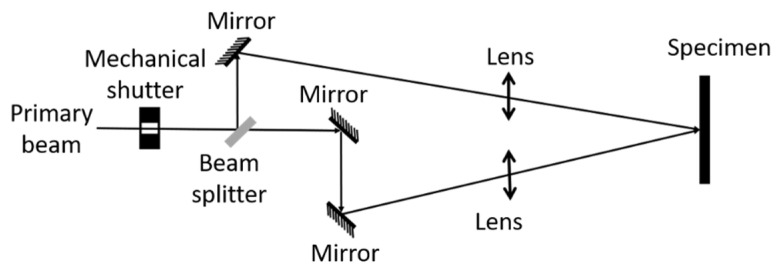
Schematic diagram of the laser-interference system. An angle between the beams of α=12° is used in this study.

A 10-Hz Q-switched Nd:YAG laser (Quanta-Ray PRO230; Spectra Physics) was used as the laser source [14,23]. The laser pulse duration was 8–10 ns. The laser pulse fluence (pulse energy per unit area) was increased by using two identical focal lenses in each path of the beams to focus them to either 4 mm or 5 mm spot size (for which 1/e^2^ beam radius was 2 or 2.5 mm, respectively) from its original beam size of 8 mm [17,23]. The emission wavelength of 1064 nm was transformed to 355 nm using non-linear crystals, decreasing the pulse duration to 8 ns. At an average power of 3.5 W, which was measured with a power meter at 355 nm, this would yield a peak power of ~43.7 MW, resulting in very high heating and cooling rates. The number of pulses was selected using a mechanical shutter. The power profile was Gaussian. The laser-interference power profile was created by splitting the beam and guiding those split beams to the sample surface by overlapping them with pre-defined angles with respect to each other (Figure 1). The coherent beams created an interference pattern instead of just adding their intensity. This allowed a microscopic modulation and created a light pattern without loss of energy during the interference process. The periodicity between power peaks and laser-interference-induced undulations is defined by the wavelength, λ, and the angle, α, between the two beams, as d = λ/2(sin α/2). A short review on physical phenomena that yield laser-interference structuring is provided in the next paragraph to better understand the surface preparation considered in this study.

When the laser reaches the rough surface of the metal, multiple reflections due to the roughness increases the absorption coefficient. Absorbed photons are instantaneously transformed into heat, which causes a temperature rise in the metal. If the temperature of the metal exceeds its melting point or saturation temperature at environmental pressure, the metal will melt or evaporate, respectively, and induce surface structuring [18]. Lasagni et al. [14] studied laser-interference structuring of metals using steel, Cu, and Al by both simulation and experiment. Numerical simulations were conducted considering the energy required for melting and vaporization. Three critical parameters were identified that govern the type of surface morphology based on the power interference distribution on the metal surface: (1) the thermal diffusion length; (2) the thermal gradient from maximum to minimum power distribution; and (3) the surface flow of the molten metal. It was concluded that thermal gradient between the maxima and minima interference induced the surface tension gradient and flow in the molten metal. The surface structure was produced by the molten metal flow [15,18].

### 2.2. Process Parameters for Laser Surface Preparation

In order to study the effects of laser-interference structuring on copper 110, several different processing methods and parameters were controlled. There were two different processing methods explored: spot-by-spot and laser raster. The spot-by-spot method used the mechanical shutter along with a moving platform. The shutter opened and closed to control the number of pulses fired that hit on the same location of the surface. After one spot was finished, the platform that holds the specimen moved to the next spot and repeated the laser structure, eventually processing the whole area. For the same spot, a higher number of pulses resulted in a higher input energy and may have caused a different surface profile. The spot-by-spot method provided a precise control over the number of laser pulses for each spot. For the laser raster method, the mechanical shutter stayed open the entire time while the specimen was moved by the platform on a straight line. The overlap between adjacent raster lines was 1 mm. There was no separately processed spot, but rather a continuously processed region. The raster speed determined the energy applied on the surface. A slower raster speed resulted in a higher input energy per area. Comparing the two methods, the main difference is whether the laser is moving on the surface. The spot-by-spot method, therefore, has an inherently slower processing speed compared to the raster method.

In this work, two laser fluences of *F*_1_ = 1.782 and 2.785 J/cm^2^ per pulse were used by varying the laser spot size (*d_b_* = 5 and 4 mm, respectively) while keeping the same average power of 3.5 W. In the experiments, 2, 4, 6, 8, 10, and 12 pulses per spot were used for the spot-by-spot method and 2, 4, 6, 8, 10, and 12 mm/s raster speeds were used for the laser raster method. Another crucial parameter was the laser spot size, which determined the energy density of each pulse on the surface. Based on the melting point of the copper and the absorbance of the surface, 4 mm and 5 mm spot sizes were selected for the spot-by-spot method [23]. For the laser raster method, only a 5 mm spot size was used, based on the results acquired for the spot-by-spot processing method. For rastering with a 5 mm spot size and a 1 mm overlap between raster lines, the Gaussian power intensity would vary between I_max_/2 and I_max_. The beam angle was 12° for an optical theoretical periodicity of the structuring of ~1.7 μm.

To understand the effect of the rastering speed, U, of the laser beam on the energy deposited on the specimen surface, two process variables were introduced: (a) the number of equivalent pulses, *N_P_*, and (b) the accumulated fluence on specimen surface, *F_A_*. *N_P_* represents the number of pulses that a local area is exposed, $N_{P}\left(U\right)=\frac{d_{b}\cdot f_{L}}{U}$, where $f_{L}$ is the laser frequency. *F_A_* represents the total incident laser energy that a local area would be exposed to from the total *N_P_* pulses striking it as the laser beam is scanned over it, as *F_A_ = N_P_ F*_1_, where the fluence of each shot is *F*_1_. The specimen labels, number of equivalent pulses, $N_{P}$, and the accumulated fluence on specimen surface, *F_A_*, are given in Table A1 and Table A2 for all of the laser structuring conditions considered in this study. As the raster speed is increased, the surface is exposed to a smaller number of shots and smaller accumulated fluences.

Among all of these various processing methods and parameters, there were 18 different processing conditions. For each condition, two different specimens were processed, resulting in a total of 36 processed specimens. The detailed process parameters with specimen labels are shown in Table A1 and Table A2 for the spot-by-spot and raster methods, respectively (Appendix A).

**Figure 2:**
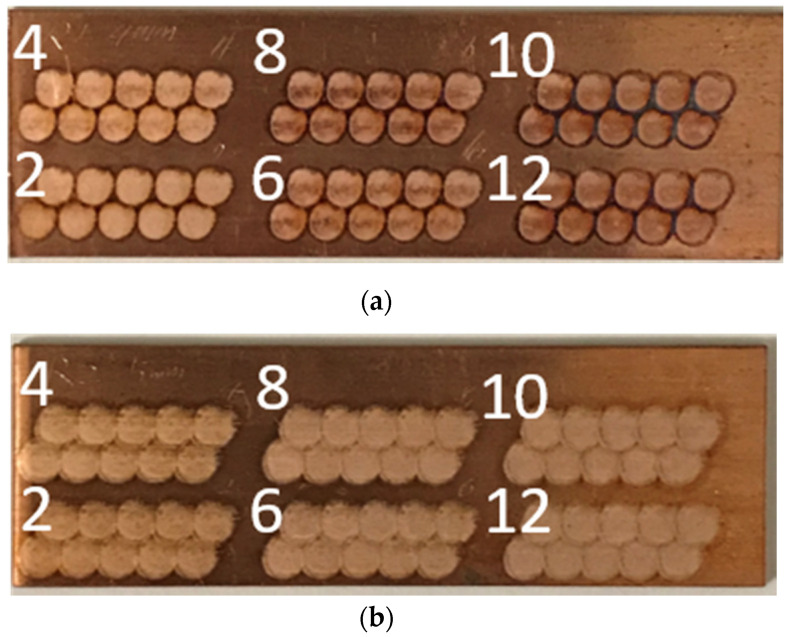
Photographs of the laser-processed area on copper using the spot-by-spot method with laser spot sizes of: (**a**) 4 mm and (**b**) 5 mm. The photos were taken immediately after processing. The label numbers correspond to conditions shown in Table A1.

As shown from the images of the processed samples in Figure 2, the processed area has a visibly different reflective color and roughness compared to the original surface. The edge of each spot can be clearly identified. Note that the edges of the spots overlap by design in order to eliminate unprocessed areas between the spots. Compared to the 5 mm spots, which blend together in color over the processed area, the 4 mm spot edges changed to a dark color, even for the lowest two pulses per spot. This indicates that the energy per pulse is sufficiently high that the Cu reacted with the oxygen in the air while processing. This became more obvious with higher number of pulses per spot. This indicates that the energy density for the 4 mm spot size was too high, and subsequent surface structuring using the laser raster method only considered 5 mm spot sizes.

### 2.3. Analysis of Surface Topology Periodicity Induced by Laser-Interference

The theoretical optical periodicity value of 1.7 μm is slightly lower than that of ~2 μm, which was estimated for Cu in [14] for a 10 ns laser pulse based on thermal diffusion length considerations. The minimum periodicity of the structuring, $p_{min},$ can be crudely estimated to be $p_{min}\sim 2\;L_{th}$, where the thermal diffusion length, $L_{th}$, a measure of the heating localization, can be estimated using the time-scale given by the pulse duration ($\tau_{p}$) and thermal diffusivity (*α*), as $L_{th}=\sqrt{\alpha \tau_{p}}$. For an 8 ns pulse of energy and thermal diffusivities of α~11.2, 10, 9, and 8 × 10^−5^ m^2^/s that were measured for electrolytic Cu at 400, 600, 900, and 1222 K [24], the $p_{min}$ was estimated to be 1.9, 1.8, 1.7, and 1.6 μm, respectively. Thus, for the optics used, the periodicity selected in this study (~1.7 μm) is very close to the estimated minimum value based on thermal diffusion length. In addition, smaller values of maximum attainable structuring depths, $d_{max}$, are expected for smaller periodicities.

Concerning the laser fluence effect on the laser-interference induced structuring, the following considerations can be made. Based on the data in [14], for single pulse experiments on Cu at periodicities of 3.51 μm, the structuring would be evident, although at very small depths ($d_{min}$), for laser fluences of $F_{min}\sim$1.7 J/cm^2^ per pulse compared to the full structure depths ($d_{max}$) that would be attained at ~2.7 J/cm^2^ per pulse. To quantify the structuring effectiveness, Sabau et al. [25] introduced a threshold fluence, $F_{th}$, for which the structure depth would be approximately half that of the maximum, or $d\sim 0.5d_{max}$. For $F_{1}\geq F_{th}$, ‘effective’ structuring characterized as $0.5d_{max}\leq d\leq d_{max}$ would be attained. At *p* = 3.51 μm, the threshold $F_{th}$ is ~2.15 J/cm^2^, as summarized in Table 1 We note that the Cu surfaces used in [20] were ground and polished with diamond suspensions while the Cu surfaces used in this study were cold-rolled. For aluminum, D’Alessandria et al. [16] found that the interference structuring was attained at lower fluences for relatively rougher surfaces and larger periodicities. Using the trends in data [16], both the $F_{min}$ and $F_{th}$ may be considered to shift from 1.7 and 2.7 J/cm^2^ for polished Cu surfaces to 1.3 and 1.72 J/cm^2^ for rough Cu surfaces. This was explained by the intrinsic smaller reflectivity for rougher surfaces than that for the polished surfaces. In this work, two laser fluences of *F*_1_ = 1.782 and 2.785 J/cm^2^ per pulse were used by varying the laser spot size (*d_b_* = 5 and 4 mm, respectively) while keeping the same average power of 3.5 W. In summary, considering these threshold fluences for polished surfaces, roughness effect, and the small periodicity selected (~1.7 μm), the use of the *F*_1_ = 1.782 and 2.785 J/cm^2^ per pulse seems to cover two possible laser-interference structuring domains, namely, the former with minor structuring and the latter with effective structuring. However, the quality of the structuring is expected to be affected by the small periodicity selected.

### 2.4. SEM Characterization

A HITACHI S-4800 scanning electron microscope featuring a maximum resolution of 1.0 nm and a variable acceleration voltage of 0.5–30 kV was used. All of the images shown in this study used a 20 kV acceleration voltage, and five different magnifications were used (300×, 500×, 1000×, 2000× and 5000×).

### 2.5. Profilometry Characterization

A Wyko NT 9100 optical profiling system was used to acquire the surface profile data at a magnification of 50×. The area chosen for surface profiling was located near the spot center for spot-by-spot processing and near the centerline of a laser scan for the raster processing. The average roughness, or arithmetical mean deviation of the roughness profile, R_a_, was calculated from the profile measurements. R_a_ was calculated by averaging the absolute height variation within the sampling area by excluding a few outlying points so that the extreme points had no significant impact on the final results.

### 2.6. Joining Procedure

Single-lap shear joints were made and tested, using both a traditional mechanical/chemical surface preparation method and laser-interference structuring to study the bonding strength. For baseline joints, a traditional mechanical/chemical surface preparation method recommended by the adhesive manufacture was applied. The Cu surfaces were first wiped clean with by IPA, followed by sanding using 220 grit sandpaper. After sanding, the surfaces were cleaned using IPA again. For the laser-structured joints, the Cu surfaces were processed using the interference technique in as-received condition, i.e., without any solvent wiping. After the surface preparation, specimens were stored in plastic cases (under ambient conditions) and bonded within 48 h of the structuring. The laser-structured surfaces were not in direct contact with any other surfaces at any time to minimize airborne contamination.

**Figure 3:**
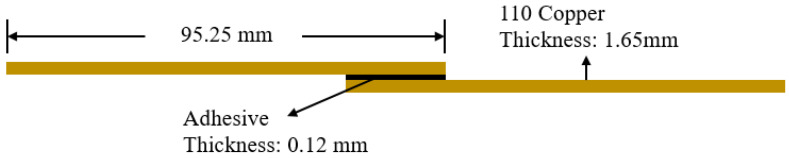
Schematic figure for the single-lap shear joints.

**Figure 4:**
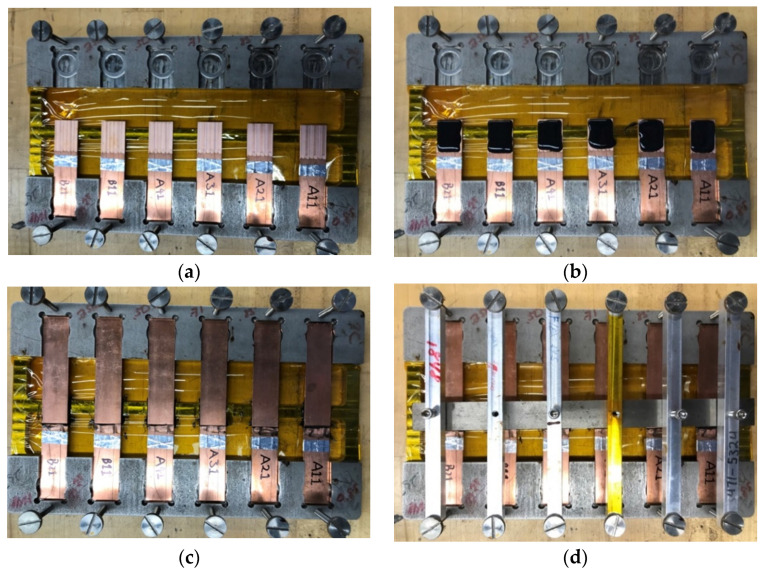
Photographs showing the single-lap shear join bonding process and fixture: (**a**) specimens placed on one side of the fixture; (**b**) adhesive applied on the bonding area; (**c**) specimens placed on the other side of the fixture; (**d**) tightened bolts pressed the two specimens firmly onto each other.

The single-lap shear joints were bonded with specially designed fixtures according to the ASTM D1002-10 standard. The Cu specimens were all cut into 25.4 mm (1 in.) × 95.25 mm (3.75 in.) sizes from the same 110 copper sheet having 1.65 mm (0.065 in.) thickness. The bonding overlap length was controlled to 12.7 mm (0.5 in.), as shown in Figure 3. Two specimens were bonded using a toughened, two-part epoxy structural adhesive (3M^TM^ Scotch-Weld^TM^ Epoxy Adhesive DP420, 3M, Saint Paul, MN, USA). The adhesive bond line thickness was controlled to 0.12 mm, as recommended by the manufacturer, by laying down two fishing lines with a given diameter. The specimen alignment and overlap length were assured by the bonding fixture. The fixture has the capacity to bond eight joint samples at the same time. The first specimen was put in one side of the fixture, as shown in Figure 4a. Adhesive was evenly distributed on the bonding surface with the dispenser specified by the adhesive manufacturer. A picture of the specimens with adhesive on top is shown in Figure 4b. Extra spacers were used to keep the two bonding surfaces parallel to each other. With the spacers in place, specimens were put into the other side of the fixture, as shown in Figure 4c. These second specimens were then compressed firmly onto the adhesive using the aluminum bars on top of each sample with tightening bolts. The whole fixture with samples in place, as shown in Figure 4d, was then left at room temperature for 24 h for curing. The overlap area was approximately 25.4 mm (1 in.) by 12.7 mm (0.5 in.), following the ASTM D1002-10 standard, to ensure the specimens could be pulled apart without breaking the Cu adherents.

### 2.7. Single-Shear-Lap Testing Procedure

The tensile shear test was conducted according to the ASTM D1002-10 standard. The specimens were placed in the grips of the tensile testing machine (Instron) so that 25.4 mm (1 in.) on each end were in contact with the jaws and the long axis of the test specimen aligned with the force applied by the grips. The load was applied to the specimen until failure with an increasing quasi-steady displacement rate of 0.5 mm/s. The force and displacement curves output from the tensile testing machine, length and width of the specimen, and overlap distance of the joints were measured for each specimen. A visual inspection was performed after the test to identify the failure mode of the adhesive bond.

## 3. Results

The characterization of surface topology using SEM and profilometry is first presented. Then, results for mechanical testing of single-lap shear joints are presented.

### 3.1. SEM Characterization of Surface Topology for As-Received, Unprocessed Specimen 

**Figure 5:**
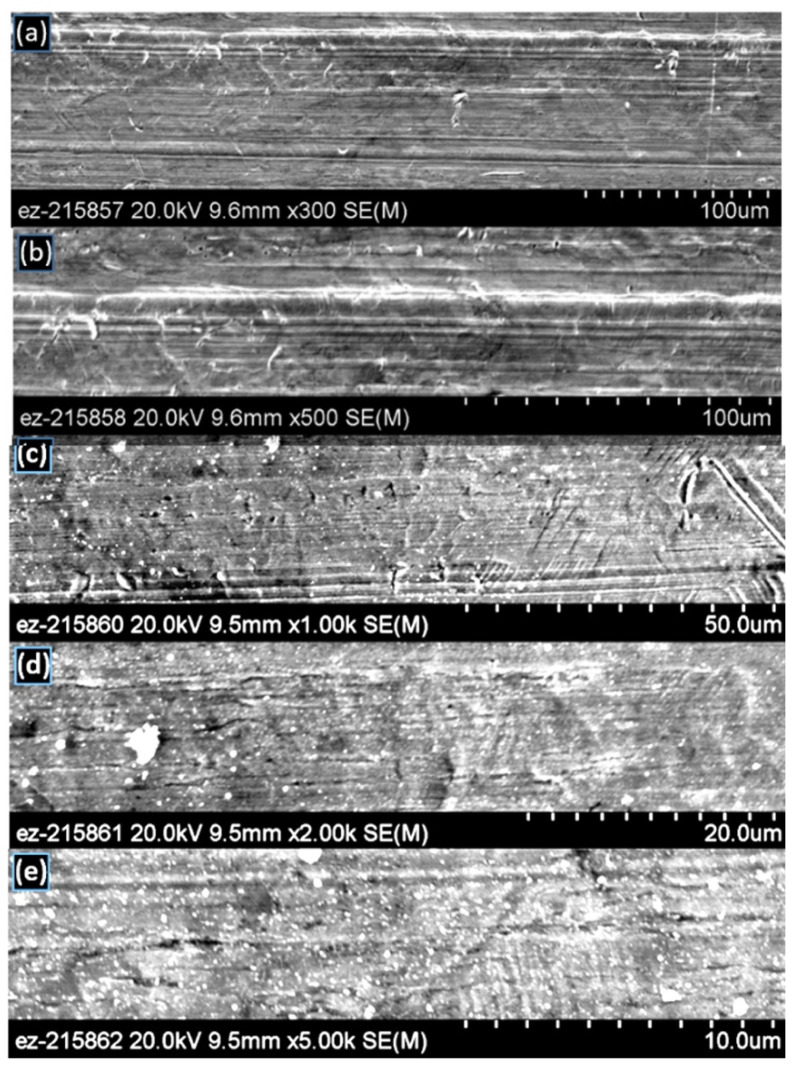
SEM micrographs of the as-received unprocessed copper surface at different magnifications: (**a**) 300×, (**b**) 500×, (**c**) 1000×, (**d**) 2000×, (**e**) 5000×.

SEM micrographs for the as-received surface are shown at different magnifications in Figure 5. The as-received surface exhibits horizontal rolling marks, many micro-cracks throughout the surface, and other surface defects. Even at a very high magnification at 5000×, the surface appears quite flat.

### 3.2. SEM Characterization of Surface Topology for Spot-by-Spot Technique

**Figure 6:**
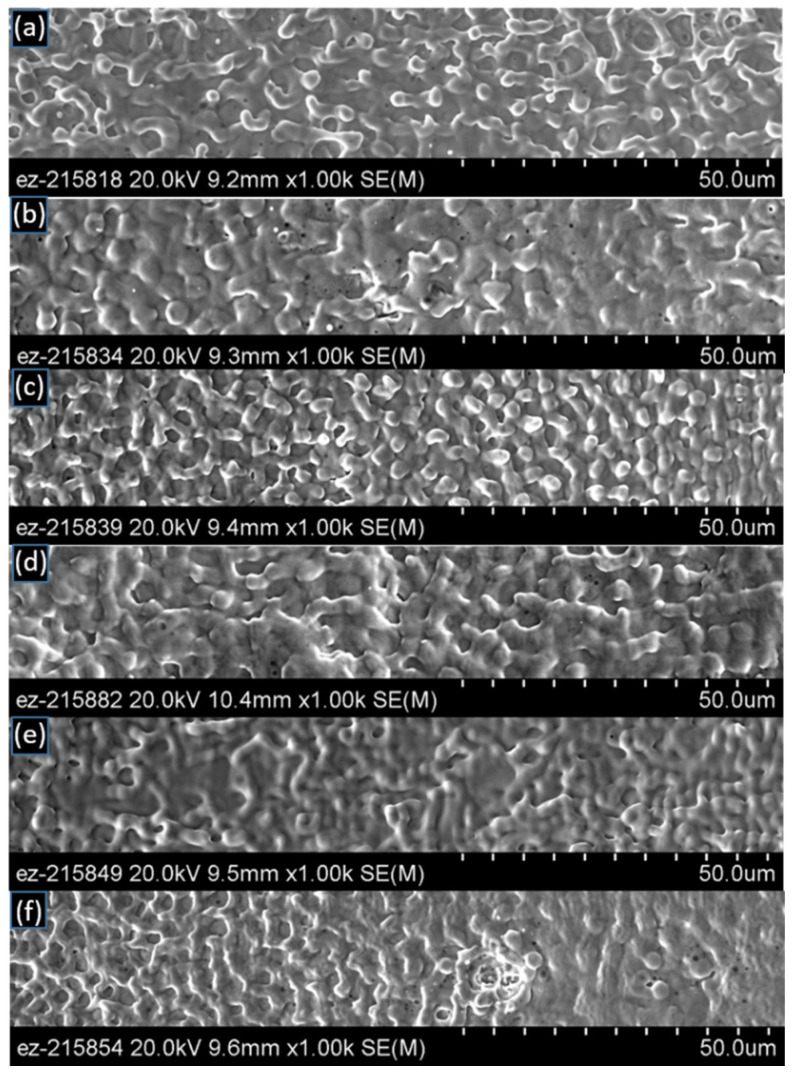
SEM micrographs (2000×) of the laser-interference-processed area using the spot-by-spot method with a 4 mm spot size on copper. Each frame shows a different number of pulses per spot: (**a**) 2, (**b**) 4, (**c**) 6, (**d**) 8, (**e**) 10, (**f**) 12.

**Figure 7:**
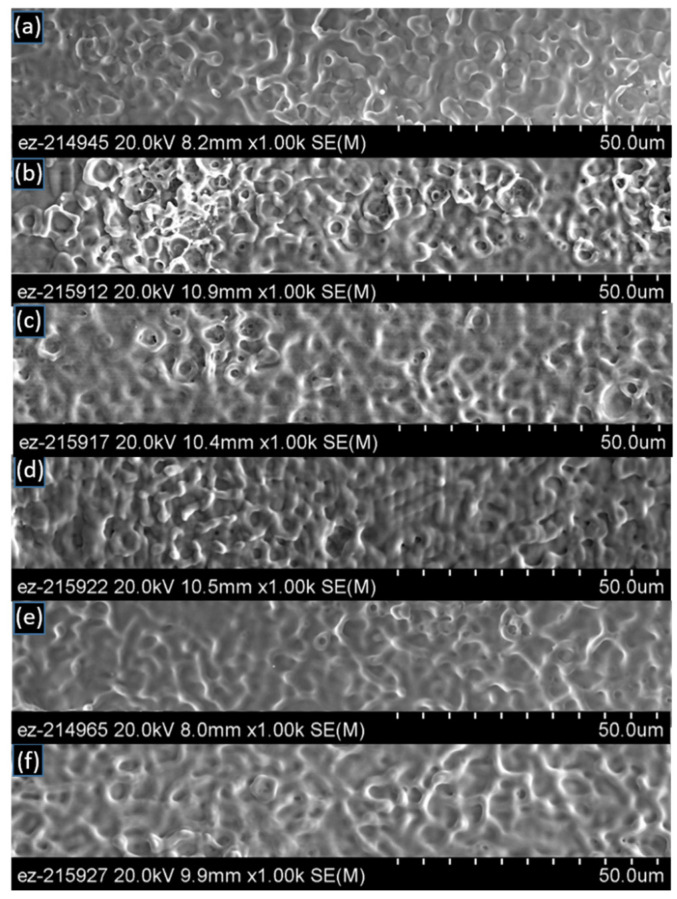
SEM micrographs (2000×) of the laser-interference-processed area using the spot-by-spot method with a 5 mm spot size on copper. Each frame shows a different number of pulses per spot: (**a**) 2, (**b**) 4, (**c**) 6, (**d**) 8, (**e**) 10, (**f**) 12.

Figure 6 and Figure 7 show the SEM images at 2000× magnification for the laser-interference-processed surface using the spot-by-spot method with 4 mm and 5 mm laser spot sizes, respectively. For the case of a 4 mm spot size, two pulses per spot already provided enough energy to form the finger-like protrusions, filament/network-like patterns that are evidence of widespread melting (Figure 6a). These types of filament/network-like topologies are a result of melting not only at the interference maxima but also at the interference minima. It is likely that the interference profile had a role to play in the formation of this type of fingering. In Table 2, an attempt was made to describe the surface topology qualitatively by indicating the density of geometrical features (i.e., protrusions), their height, and their characteristic size. Further increasing the number of pulses seems to alter the type of net-like geometrical features (Table 2). Larger surface areas are considered to be more beneficial for bonding. Large surface areas would result from taller features and higher density of features per unit surface area. The height and density of these melt-induced features appear to be maximized for two and six shots per spot. For these cases, the accumulated fluences on specimen surfaces were 3.56 J/cm^2^ and 7.13 J/cm^2^, respectively. The height and density of these melt-induced features seems to be minimized for specimens with 10 and 12 shots per spot.

The surface topology is shown in Figure 7 for the 5 mm spot size cases. For 5 mm spot size and two pulses per spot (Figure 5a); surface topologies seemed to be similar to those observed for the case with the 4 mm spot size and two pulses per shot (Figure 6a). When four pulses per spot were used (Figure 7b), melt rings and microcrater-like features appeared. Further increasing the number of pulses did not appear to alter the characteristic size of geometrical features (Table 3), which, on average, was ~1.6 μm for all conditions, while for the 4 mm spot size, larger features were observed, as large as 4.5 μm. In general, fewer finger-like protrusions were evidenced for the 5 mm spot size than for the 4 mm beam size specimens (e.g., Figure 5d and Figure 7a versus Figure 4c,d and Figure 6a) such that the net-like surface topology characterized the 5 mm spot processing. Additionally, the density of geometrical features, e.g., protrusions, did not seem to vary with the number of pulses per spot, while a wider range of variation was observed for the 4 mm beam size specimens. For the 5 mm spot size, the surface was flatter for 10 and 12 pulses per spot than for all the other conditions.

Since the expected periodicity was not attained, it is important to investigate what type of surface topology would be attained without laser-interference, i.e., using the main laser beam only without splitting it. The surface topology for specimens processed without laser-interference is presented in Appendix B, for similar laser parameters to those for which the surface topology is discussed in this Section. The data shows that the laser-interference yields surface topologies with finer protrusions and more uniform surface topologies than processing without laser-interference (Appendix A).

**Figure 8:**
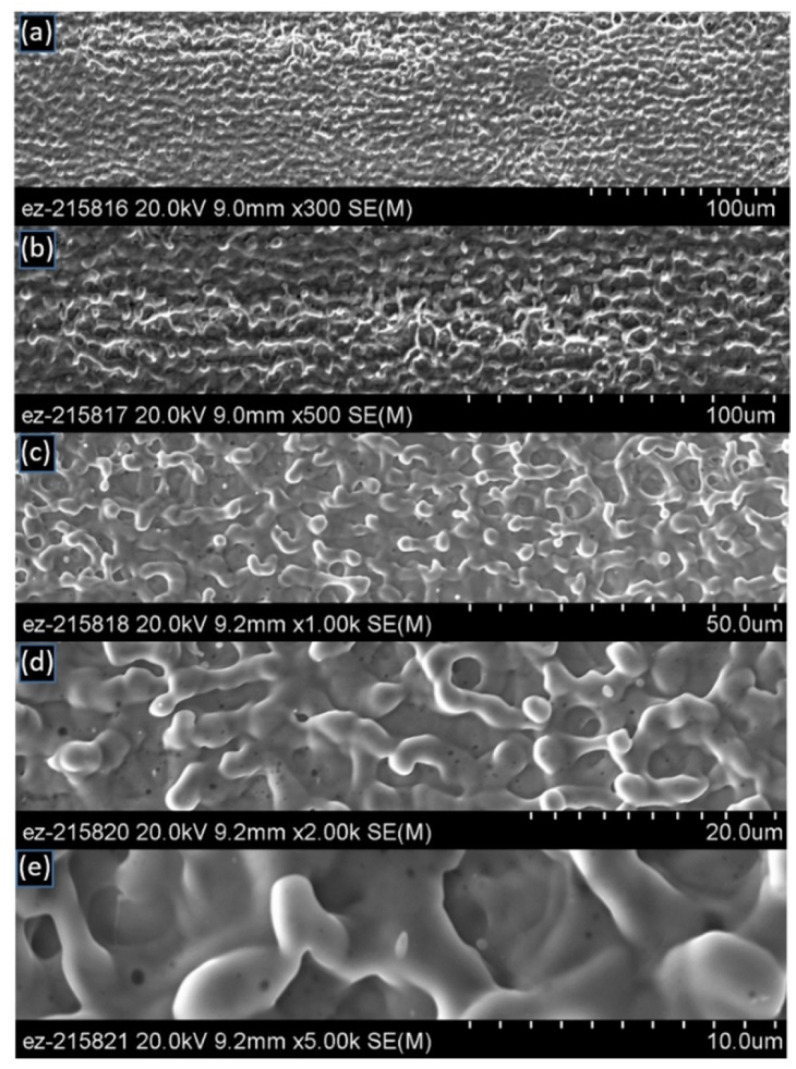
SEM micrographs of the laser-interference processed Cu surface (spot-by-spot method) with a 4 mm spot size and two pulses per spot at different magnifications: (**a**) 300×, (**b**) 500×, (**c**) 1000×, (**d**) 2000×, (**e**) 5000×.

For the sake of completion, a montage with SEM micrographs taken at similar magnifications as those for the as-received specimen (Figure 5) was assembled for one laser-processed specimen with 4 mm spot size and two pulses per spot in Figure 8. At higher magnification, as shown in Figure 6e and Figure 8d, the filament web diameter size was measured to be ~2 μm.

### 3.3. SEM Characterization of Surface Topology for Raster Technique

**Figure 9:**
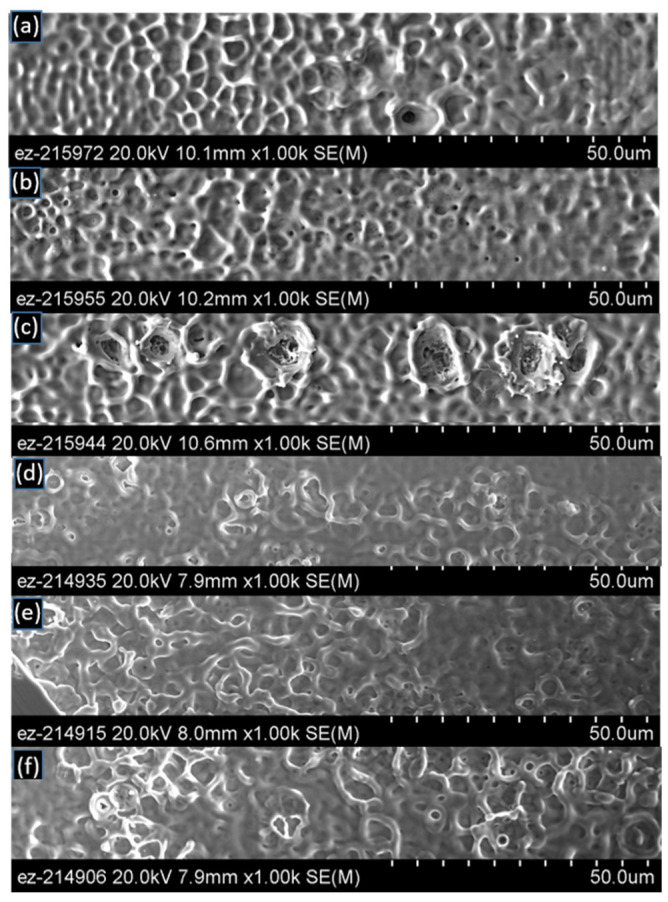
SEM images (2000× magnification) of the laser-interference-processed area using the laser raster method with a 5 mm spot size on copper. Each frame shows a different raster speed (mm/s): (**a**) 2, (**b**) 4, (**c**) 6, (**d**) 8, (**e**) 10, (**f**) 12.

After spot-by-spot processing, the laser raster processing was conducted for only the 5 mm laser spot size. Six different raster speeds were used: 2, 4, 6, 8, 10, and 12 mm/s. In the raster method, a higher speed decreased the overall input energy. The resulting surface topologies are shown in Figure 9 at a SEM magnification of 2000×. At lower raster speeds of 2 and 4 mm/s (Figure 7a,b), the raster method was found to yield similar surface topologies to those from the spot-by-spot method. In Figure 9a, for the lowest speed, the net-like structure could be found in the left side of the SEM with a flatter uniform right side. Crater-like features were evidenced for the 4 mm/s case (Figure 9b), similar to those for the four/six pulses per spot for the spot-by-spot processing (Figure 6c and Figure 8b). As the raster speed was increased from 6 mm/s to 12 mm/s (Figure 9c through Figure 7f), the surface topology became less regular. A summary of surface topologies for the raster technique is presented in Table 4. In general, the surface topology appeared to be flatter than that for the spot-by-spot method with thinner net-like protrusions sticking out of the surface.

### 3.4. Surface Profile

**Figure 10:**
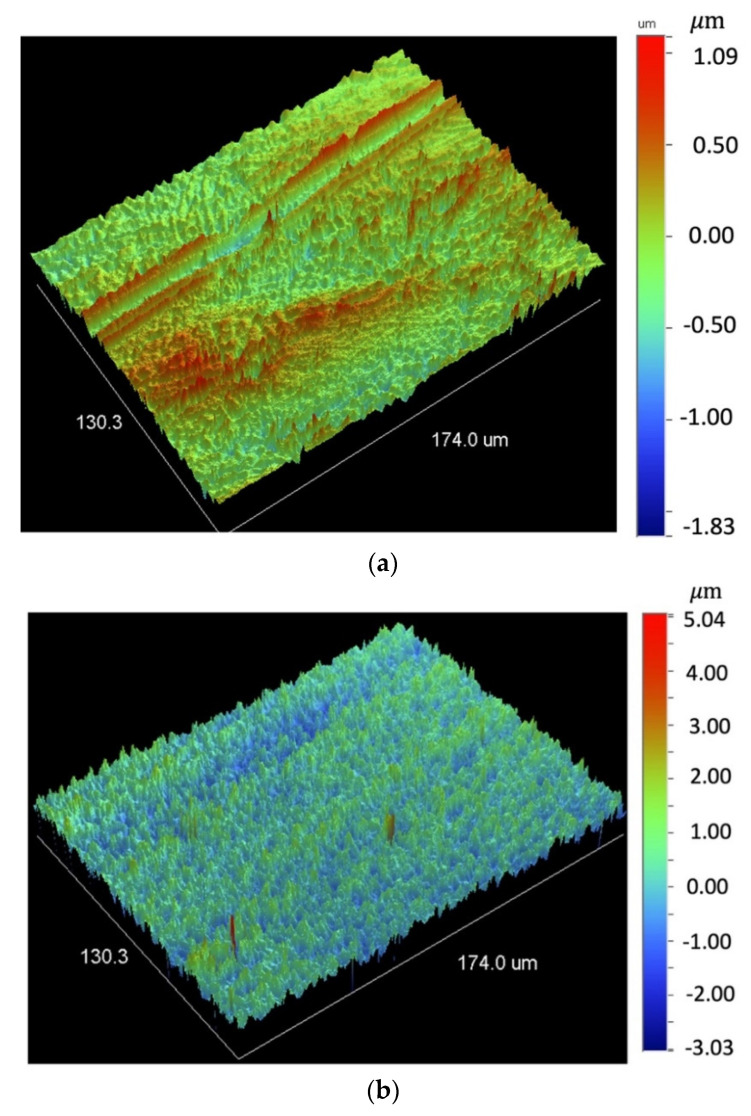

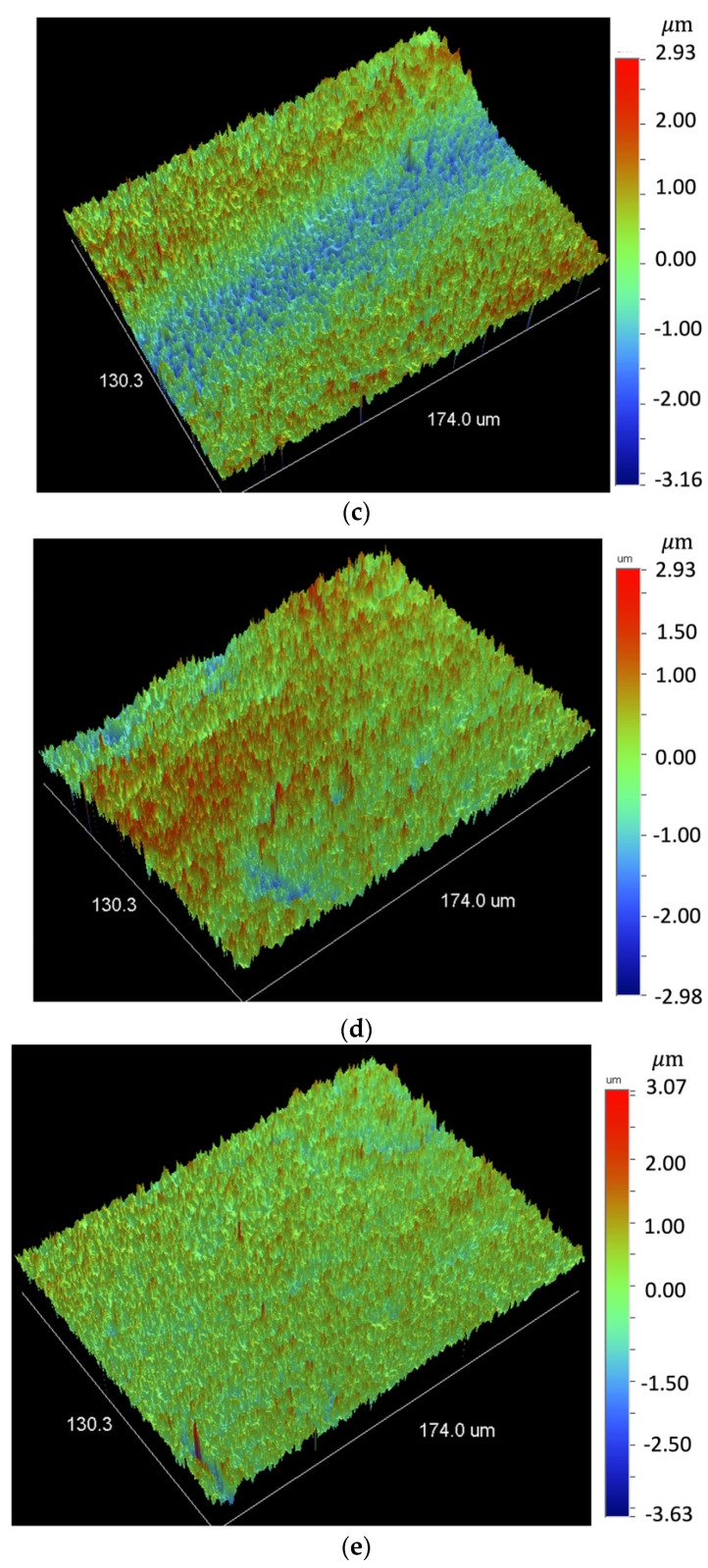
3D surface profile at 50× magnification for: (**a**) as-received, unprocessed specimen and (**b**–**e**) spot-by-spot laser-interference processed Cu: (**b**) two pulses *F*_1_ = 2.785 J/cm^2^ per pulse, (**c**) two pulses *F*_1_ = 1.782 J/cm^2^ per pulse, (**d**) six pulses *F*_1_ = 1.782 J/cm^2^ per pulse, and (**e**) 12 pulses *F*_1_ = 1.782 J/cm^2^ per pulse.

**Figure 11:**
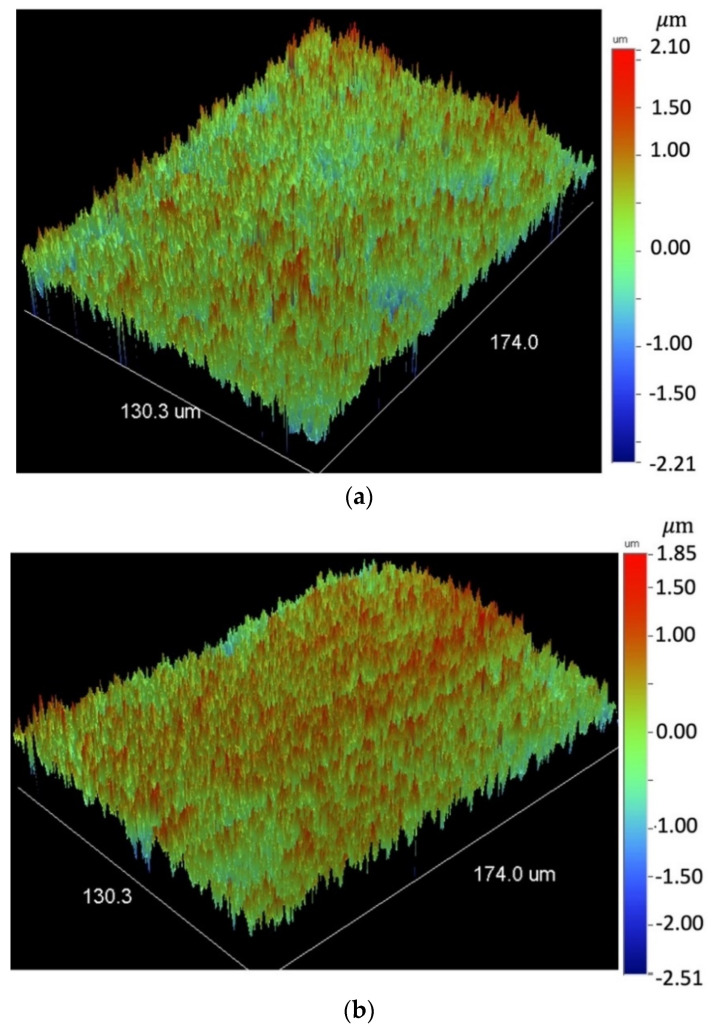
3D surface profile at 50× magnification for laser-interference raster processed with *F*_1_ = 2.785 J/cm^2^ per pulse (5 mm spot size): (**a**) 2 mm/s and (**b**) 6 mm/s.

Figure 10a shows one surface profile for the original surface in the as-received, unprocessed condition. In the surface profile, grooves from the prior rolling operation are present without any other patterns. The surface profile for the spot-by-spot laser-processed specimen, for which the SEM micrographs were shown at different magnifications in Figure 6, is shown in Figure 10b. The surface profile appears to be much rougher than that for the as-received condition. The surface profile for two raster processed specimens were shown in Figure 10c,d. The R_a_ was estimated to be 183 nm for the unprocessed specimen. For two pulses/spot at a laser fluence of *F*_1_ = 2.785 J/cm^2^ per pulse (4 mm spot size), R_a_ was 394 nm (Figure 10b). For a laser fluence of *F*_1_ = 1.782 J/cm^2^ per pulse (5 mm spot size), R_a_ was 329, 407, and 585 nm for 2, 6, and 12 pulses/spot, respectively (Figure 10c–e). Thus, R_a_ was doubled and tripled, respectively, by laser spot-by-spot processing for laser fluences of *F*_1_ = 2.785 and 1.782 J/cm^2^ per pulse. For a laser fluences of *F*_1_ = 1.782 J/cm^2^ per pulse (5 mm spot size), R_a_ was 340, and 364 nm for raster speeds of 2 mm/s and 6 mm/s (Figure 11a,b), respectively. Basically, R_a_ was doubled by laser raster processing. A chemical analysis to assess the surface contamination was not conducted in this study as this effect is very well documented in the literature for a single laser beam [13], as well as for laser-interference [23,25].

### 3.5. Joint Strength Evaluation

In this section, results for the single-lap shear joints, which were made with a traditional mechanical/chemical surface preparation method and laser-interference structuring, are presented. The raster processing method was selected to prepare surfaces for joining. The raster method was selected based on several considerations. First, compared with the spot-by-spot method, as shown in Figure 5 and Figure 6, the laser raster method resulted in net-shape structures that had a smaller height and finer length scale. Second, the laser raster method is faster than the spot-by-spot method. For an industrial application, the two techniques would be actually merged by using: (a) a laser fluence (i.e., laser spot size) that would ensure appropriate texturing in one pulse and (b) a raster speed that would move the beam adjacent to the prior spot for the next pulse.

As analyzed in Section 3.4, the laser raster method using a 5 mm spot size beam was selected. Considering the range of surface profiles attained, the 6 mm/s and 12 mm/s raster speeds were selected to ensure distinct characteristics between each sample. The specimen types were labeled as follows: A for baseline, B for LIS with 6 mm/s, and C for LIS with 12 mm/s. All of the other laser parameters were kept the same as those in the surface characterization study. Three specimens were prepared for the mechanical/chemical surface preparation baseline, and four specimens were prepared for each laser raster condition.

### 3.6. Single-Lap Shear Strength Results

**Figure 12:**
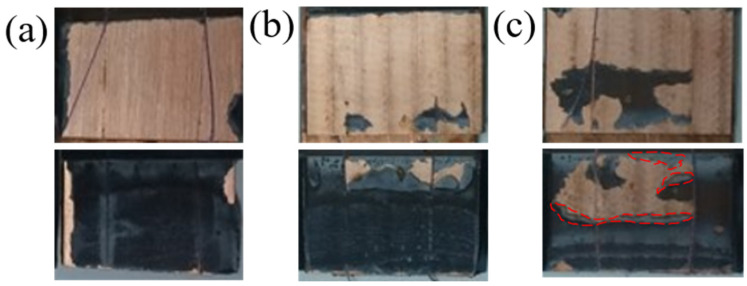
Selected photographs of the overlapping bonding area after single-lap shear testing. Each top and bottom pair of images correspond to the same bonded specimen: (**a**) A2 baseline specimen; mixed failure, (**b**) C3 (12 mm/s) adhesive failure; and (**c**) C4 (12 mm/s) adhesive failure.

In this section, the results of the adhesive joint shear strength are analyzed and discussed. The failure mechanism can be identified by inspecting the adhesive and adherent surfaces in the overlapping region. Selected photographs of these interfaces are shown in Figure 12. For the cases shown in Figure 12a, most of the adhesive was found attached to one adherent while the other adherent had little trace of the adhesive. This indicates that an ‘adhesive’ failure occurred at the interface between the adherent and adhesive, as opposed to a ‘cohesive’ failure inside the adhesive material itself. In Figure 12b, adhesive remnants can be found on both adherents; however, if matched back together, it is clear that the adhesive left on both surfaces has a small overlapping region. The overlapping fracture layer can be seen as the lighter gray regions outlined in red in the bottom image. This indicates a ‘mixed’ failure of the adhesive, with peeling of the adherents during the test. These adhesive and mixed failure mechanisms are not common for toughened epoxy adhesive and are attributed to the nature of the single-lap joint test. Although the specimens were cut from the same material lot and carefully aligned along the tester axis to reduce the eccentricity of the load path, the high shear force needed to pull apart the specimens still caused out-of-plane bending moments.

The maximum loading force for single-lap shear joints is affected by several factors, including the surface preparation method, adhesive thickness, and joints geometry. In the experiments, the adhesive thickness was precisely controlled to be the same for all samples (0.12 mm). In order to isolate the effect of different surface preparation methods, the influence of the bonded area of the joint was reduced by comparing the joint shear lap strength, which was estimated by dividing the maximum load by the overlap bonding area (overlap length times the specimen width). The specimen width was uniform at 25.4 mm (1 in.), but due to slight variation in the cutting operation, the overlap length varied.

**Figure 13:**
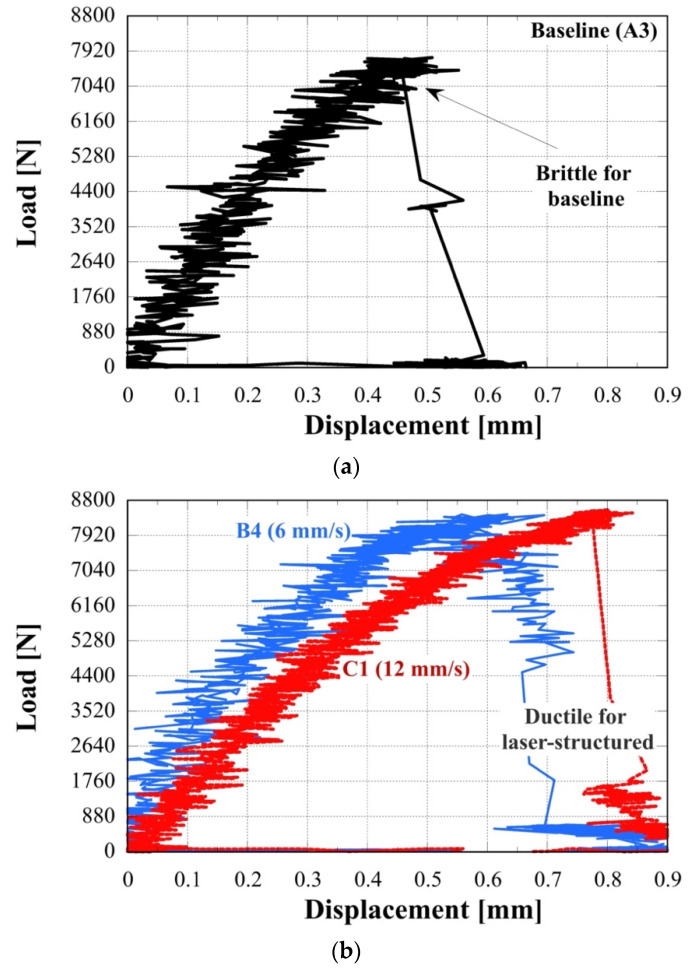
Load versus displacement variation during single-lap shear testing for (**a**): baseline specimen A3 and (**b**) laser-structured specimens B4 and C1 (6 and 12 mm/s raster speed, respectively).

The load-displacement curves are shown for several specimens in Figure 13. For the baseline specimen, the variation of the load versus displacement curve near the failure point was characteristic of a brittle fracture at failure; for the laser-structured specimens, a ductile fracture was observed, i.e., failure point after a slow decrease in the load rate. These data indicate that the laser-structured joints are more ductile than those without laser-structuring. The increased ductility of the laser-structured joints indicates an enhanced bonding of the adhesive to the Cu adherents. The energy absorbed by the joint during the deformation testing, which is proportional to the area under the load versus displacement curve, is another parameter that can be used to quantify the joint performance. The energy absorbed during the tensile pull was calculated for the data shown in Figure 13 to be 2.02, 3.71, and 3.89 Joules for the baseline joint and laser-structured joints with 6 mm/s and 12 mm/s, respectively. Thus, the absorbed energy during shear-lap testing of laser-structured joints was approximately 1.8 to 1.9× than that for the baseline specimen, i.e., an increase by approximately 80–90% over those measured for baseline joints.

The results of the tensile shear testing are shown in Table 5. The results for specimen C4 exhibit a significant lower maximum loading, shear stress, and maximum displacement than the other samples.

**Figure 14:**
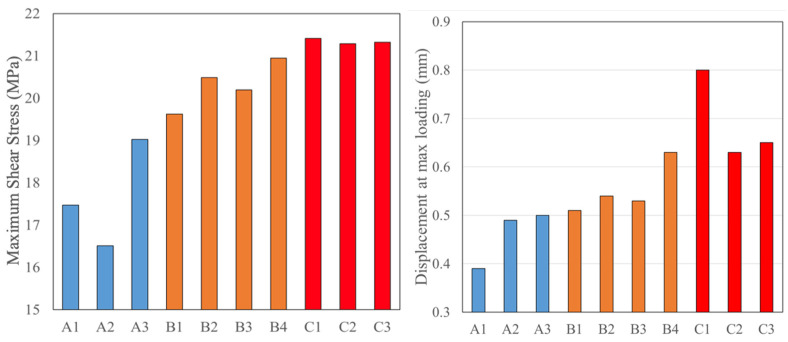
Shear strength (**left**) and displacement at maximum loading (**right**) for the laser-structured specimens and baseline (A: Baseline; B: 6 mm/s; C: 12 mm/s).

The shear strength and displacement at maximum loading for the rest of the specimens have a clear trend that can be seen in Figure 14. The strength and displacement at maximum load are both increased for the laser-structured samples compared to those surfaces prepared by traditional methods.

The statistics metrics were evaluated using the shear-lap testing data for all the specimens, which is shown in Table 5 and shown in Table 6. As shown in Table 6, compared with the traditional method, surface preparation with a laser raster at 6 mm/s was found to increase the shear strength and displacement at maximum loading by 11% and 25%, respectively. At 12 mm/s, the increases in the shear strength and displacement at maximum loading were 17% and 44%, respectively. These shear-lap testing results indicate that the laser structuring with 12 mm/s yields better shear-lap joints than the laser structuring with 6 mm/s. As shown in the remainder of this paragraph, this finding is in good agreement with the mechanical interlocking and adhesion expected for the surface morphology for these conditions. Basically, the 12 mm/s laser processing was shown to yield increased protrusion heights over those with the 6 mm/s, at similar protrusion feature density (Figure 9c,f, and Table 4). Thus, a larger surface area is expected for the 12 mm/s processing than for the 6 mm/s processing. In turn, increased surface areas would enhance the mechanical interlocking and adhesion, yielding higher shear strengths. However, detailed explanation for the higher raster speed causing a larger enhancement shown in Table 6 needs further testing and surface characterization, such as XPS and wettability tests, to understand and quantify these factors.

## 4. Conclusions and Discussions

In this study, laser-interference structuring near the interference limit imposed by the localized energy transport was evaluated as a surface preparation technique for adhesive bonding of copper. A nanosecond pulsed Nd: YAG laser was operated at 355 nm wavelength and laser fluences of *F*_1_ = 1.782 and 2.785 J/cm^2^ per pulse. The optical setup considered would enable a structuring periodicity of 1.7 μm, which is near the 1.6–2 μm structuring limit that was estimated based on thermal diffusion lengthscale for an 8 ns laser pulse.

The surface topology of Cu formed using spot-by-spot and laser raster methods were characterized using SEM and profilometry. The SEM micrographs showed that the surface topologies of laser-processed specimens are characterized by finger-like protrusions, and filament/network-like patterns that evidence widespread melting. The characteristic size of the melt patterning, namely its height and feature density, varies with the laser conditions. These types of filament/network-like topologies are a result of melting not only at the interference maxima but also at the interference minima. Although the expected periodic interference structuring was not attained, SEM micrographs for specimens processed without laser-interference indicate that the melt-induced texturing was affected by the interference processing.

Larger surface areas are considered to be more beneficial for bonding. Large surface areas would result from taller features and higher density of features per unit surface area. The height and density of these melt-induced features appear to be maximized for two and six pulses per spot. For these cases, the accumulated fluences on specimen surfaces were 3.56 J/cm^2^ and 7.13 J/cm^2^, respectively. In general, the surface topology appears to be flatter than that for spot-by-spot method with thinner net-like protrusions sticking out of the surface. The height and density of these melt-induced features were maximized for the 12 mm/s raster processing (accumulated fluence of and 7.42 J/cm^2^). This finding for the raster method is consistent with the fact that the surface topology features were maximized for a similar accumulated fluence (7.42 J/cm^2^) to that for the spot-by-spot condition (7.13 J/cm^2^).

Joints were adhesively bonded using laser-structured specimens. Baseline joints were prepared by abrading joining specimens. Single-lap shear tests were performed for the laser-structured specimens for two raster conditions and abraded specimens. The shear-lap testing indicated significant bonding enhancement compared with a baseline preparation method. The highest raster speed yielded the best bonding performance within the test conditions investigated. The shear stress and displacement at maximum loading, at a raster speed of 12 mm/s, were increased by 16.8% and 43.8%, respectively, over those measured for the specimens prepared by the baseline method. The load-displacement curves indicated that the laser-structured joints were more ductile than those without laser-structuring. The energy absorbed by the joint during the deformation testing, which was proportional to the area under the load versus displacement curve, was found to increase by approximately 80–90% over those measured for baseline joints. The increased ductility for the laser-structured joints was another indicator of enhanced adhesive bonding. Future efforts should be focused on investigating the joining performance with laser-interference-induced surface topology at a target periodicity larger than 2 μm.

## Figures and Tables

**Table 1:** Survey of surface topology features for single pulse laser-interference processing of pure Al and Cu reported in the literatures.

*p_exp_* [μm]	$d_{max}\;[\mu m]$	$*F_{min}\;[J/{\mathrm{cm}}^{2}]$	$F_{th}\;[J/{\mathrm{cm}}^{2}]$	Metal	Surface Condition	Reference
2.2	0.7	0.9	1.2	Al	rough	D’Alessandria et al. [16]
3.4	1.4	0.97	1.37	Al	rough	
3.4	1.45	1.42	1.75	Al	polished	
4.5	1.5	0.8	0.9	Al	rough	
4.7	1.3	1.5	1.68	Al	polished	Lasagni et al. [14]
3.5	1.4	1.7	2.7	Cu	polished	Lasagni et al. [14]

*$F_{min}$ indicates the minimum fluence for which data is reported.

**Table 2:** Topology variation for spot-by-spot laser processing with 4 mm spot size.

Case No.	*N_P_*	Topology Description	Protrusion Size [μm]	Feature Density	Feature Height
1	2	Network protrusions	2	Medium	Medium
2	4	Flatter, thick network	3.5	Very low	Very small
3	6	Upward protrusions	2.5	Very high	Very high
4	8	Flatter, thick protrusions	3	Very low	Medium
5	10	Flat, thin protrusions	1.7	High	Very small
6	12	Flat protrusions	2.2–4.5	High to low	Very small

**Table 3:** Topology variation for spot-by-spot laser-interference processing with 5 mm spot size.

Case No.	*N_P_*	Topology Description	Protrusion Size [μm]	Feature Density	Feature Height
1	2	Network protrusions	2	Medium	High
2	4	Network protrusions and Melt rings	1.2–1.7	High	High
3	6	Flatter network protrusions and Melt rings	1.5	Medium	Small
4	8	Network protrusions	1.5	Medium	Medium to Small
5	10	Flatter, thin protrusions	1.5	Medium	Small
6	12	Flatter, thin protrusions	1.7	Medium	Small

**Table 4:** Topology variation for the raster laser processing with 5 mm spot size.

Case No.	*U* [mm/s]	Topology Description	Protrusion Size [μm]	Feature Density	Feature Height
1	2	Network protrusions	1.2	Medium	Medium
2	4	Network protrusions and small melt rings	2	Medium	Small
3	6	Flatter, network protrusions and large melt rings	1.5	Medium	Small/Medium
4	8	Flatter, network protrusions	1.2–1.5	Low	Small/Medium
5	10	Flatter, thicker protrusions	1.5–2	Low	Small/Medium
6	12	Deeper, thin protrusions	1.5	Medium	High

**Table 5:** Geometry, maximum loading, maximum shear stress, and displacement at maximum loading for the laser-structured (raster method) and baseline specimens (A: Baseline: B: 6 mm/s; C: 12 mm/s).

Specimen Label (Condition)	Overlap Length [mm]	Overlap Area [mm^2^]	Max Load [N]	Shear-Lap Stress [MPa]	Max Disp. at Max Load [mm]	* Surface Topology
A1 (base)	13.7	347.5	4981	14.34	0.26	Flatter, typical to rolling operations
A2 (base)	14.9	379.5	6629	17.47	0.39	
A3 (base)	16.0	407.4	7751	19.02	0.50	
B1 (6 mm/s)	16.0	406.2	7969	19.62	0.53	network, medium density, large melt rings
B2 (6 mm/s)	16.0	400.1	8194	20.49	0.51	
B3 (6 mm/s)	16.1	409.2	8263	20.19	0.51	
B4 (6 mm/s)	15.9	403.6	8453	20.94	0.63	
C1 (12 mm/s)	16.1	401.1	8563	21.34	0.80	Thin protrusions with medium density
C2 (12 mm/s)	17.1	435.1	9262	21.29	0.63	
C3 (12 mm/s)	17.0	431.6	9201	21.32	0.65	
C4 (12 mm/s)	16.1	409.7	4852	11.84	0.36	

* Topology characterization from Table 4.

**Table 6:** Statistics and percentage increase in the maximum shear stress and maximum displacement for the laser-structured specimens with respect to the baseline.

Methods	Maximum Shear Stress [MPa]	Std. Deviation [MPa]	Increase [%]	Displacement at Max Loading [mm]	Std. Deviation [mm]	Increase [%]
Baseline	18.25	1.10	N/A	0.45	0.078	N/A
Laser: 6 mm/s	20.25	0.66	11.0	0.56	0.064	25.1
Laser: 12 mm/s	21.30	0.02	16.8	0.64	0.014	43.8

**Figure A1:**
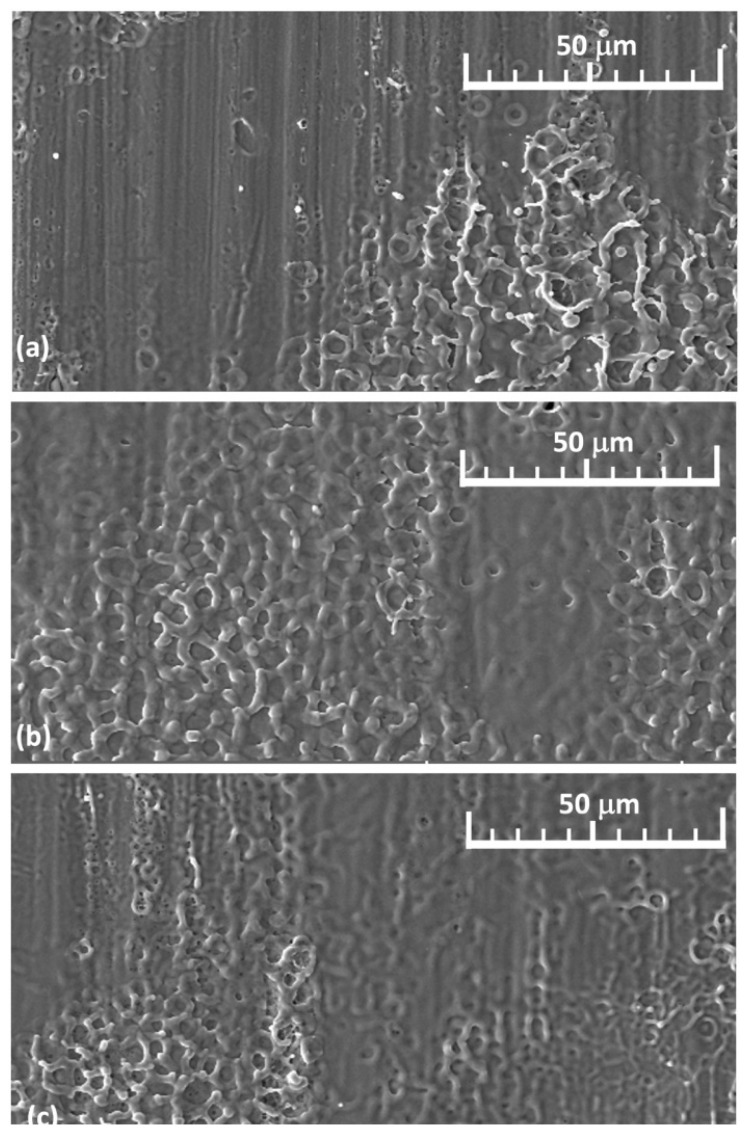
SEM micrographs for one-beam laser processed Cu specimens using the spot-by-spot method with a 5 mm spot size for number of pulses per spot of: (**a**) 2, (**b**) 4, and (**c**) 6.

**Figure A2:**
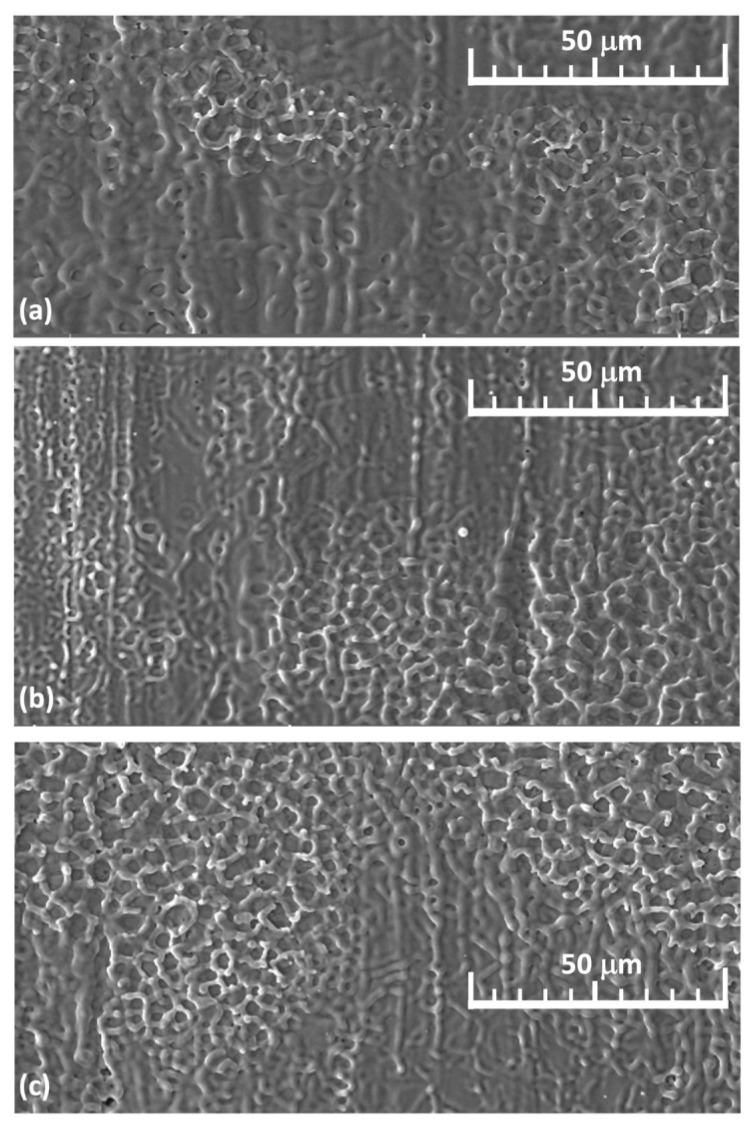
SEM micrographs for one-beam laser processed Cu specimens using the spot-by-spot method with a 5 mm spot size for number of pulses per spot of: (**a**) 8, (**b**) 10, and (**c**) 12.

## Data Availability

The data presented in this study are available on request from the corresponding author.

## Notice

This manuscript has been authored in part by UT-Battelle, LLC, under contract DE-AC05–00OR22725 with the US Department of Energy (DOE). The United States Government retains and the publisher, by accepting the article for publication, acknowledges that the United States Government retains a non-exclusive, paid-up, irrevocable, world-wide license to publish or reproduce the published form of this manuscript, or allow others to do so, for United States Government purposes. The Department of Energy will provide public access to these results of federally sponsored research in accordance with the DOE Public Access Plan (http://energy.gov/downloads/doe-public-access-plan).

## Appendix A

**Table A1:** Laser processing parameters used for the spot-by-spot method (average power 3.5 W).

Case No.	Spot Size [mm]	Number of Pulses per Spot, *N_P_*	*F_A_* * [J/cm^2^]	Figure
1	4	2	5.570	Figure 5a
2	4	4	11.14	Figure 5b
3	4	6	16.71	Figure 5c
4	4	8	22.28	Figure 5d
5	4	10	27.85	Figure 5e
6	4	12	33.42	Figure 5f
7	5	2	3.56	Figure 6a
8	5	4	7.13	Figure 6b
9	5	6	10.69	Figure 6c
10	5	8	14.25	Figure 6d
11	5	10	17.82	Figure 6e
12	5	12	21.38	Figure 6f

* *F_A_ = N_P_·F*_1_ for spot-by-spot.

**Table A2:** Laser processing parameters for the laser raster method (average power 3.5 W).

Case No.	Spot Size [mm]	Raster Speed [mm/s]	*N_P_*	*F_A_* [J/cm^2^]	Figure
13	5	2	25	44.55	Figure 7a
14	5	4	13	22.28	Figure 7b
15	5	6	8	14.85	Figure 7c
16	5	8	6	11.14	Figure 7d
17	5	10	5	8.91	Figure 7e
18	5	12	4	7.42	Figure 7f

## Appendix B

The surface topology for specimens processed without laser-interference, i.e., using only the main laser beam without splitting it, is presented in this section. The surface topology is shown in Figure A1 and Figure A2 for specimens processed with the same 5 mm spot size as for those processed with laser-interference, as shown in Figure 7. The surface topology features are summarized in Table A3. For 2, 4, and 6 pulses per spot (Figure A1a–c), the surface topologies are quite different than those with the laser-interference (Figure A1a–c), exhibiting large flat areas without protrusions. The rolling direction is evident through vertical striations in Figure A1a,b. Consequently, the feature height and protrusion size exhibit a wide range of variation for each specimen. Overall, these two, four, and six pulses per spot specimens were found to exhibit thicker protrusions than the corresponding laser-interference cases.

Concerning the appearance of the ‘less-affected’ flat areas, the following considerations can be made. First, the SEM images were taken from the laser spot center, which is a similar location to those for the laser-interference specimens. Second, as mentioned in Sabau et al. [17], for Al, the (non)uniformity of the lubrication films from prior rolling operations would affect the overall energy available locally for the laser surface treatment, the local temperature evolution during laser surface treatment, and ensuing melting. Third, the magnitude of the local laser heat flux in the interference maxima is exactly twice than that of the one-beam setup, providing more energy for the local melting than for the one-beam interference-less processing. Thus, more melting is expected for the laser-interference processing than for the traditional one-beam laser processing.

For 8, 10, and 12 pulses per spot (Figure A2a–c), surface topologies for one-beam processing seem different than those with the laser-interference processing (Figure 7d–f), exhibiting areas with small protrusion sizes (e.g., 1.2 to 1.7 μm) and areas with large protrusion sizes (e.g., 2.5 to 3 μm). The less-affected areas seen for processing with lower number of pulses are not present for the processing with a larger number of pulses. However, for 8 and 10 pulses per spot, the feature density exhibits a variation between regions with medium feature density and flatter, less dense regions. The variation in the surface topology is minimum for the 12 pulses per spot condition among all the one-beam laser specimens, making the result of this processing condition the closest to the laser-interference conditions. However, the surface topologies for the 12 pulses per spot, with and without laser-interference, still remain quite different. Based on the data presented in this Appendix for one-beam (without laser-interference) processing and corresponding data presented in Section 2.5 for the laser-interference processing at a periodicity of 1.7 μm, which is close to that estimated based on the thermal diffusion lengthscales, it can be concluded that although the laser-interference at this small periodicity does not realize the expected periodic structuring, it yields surface topologies with finer protrusions and more uniform surface topologies.

**Table A3:** Topology variation for one-beam laser processed Cu specimens with 5 mm spot size.

Case No.	*N_P_*	Topology Description	Protrusion Size [μm]	Feature Density	Feature Height
1	2	Incomplete melting; Network protrusions	0–1.9	None to Medium	None to High
2	4	Incomplete melting; Thick network protrusions	0–2.3	None to Medium	Very small to Medium
3	6	Flatter network protrusions and melt rings	0–2	None to Medium	Very Small to High
4	8	Flatter, medium and thick protrusions	1.3–3	Small to Medium	Medium to Small
5	10	Flatter, thin and thick protrusions	1.2–2.5	Small to Medium	Small
6	12	Flatter, thin protrusions	1.7–2.5	Medium	High
